# Using Large Language Models to Understand Suicidality in a Social Media–Based Taxonomy of Mental Health Disorders: Linguistic Analysis of Reddit Posts

**DOI:** 10.2196/57234

**Published:** 2024-05-16

**Authors:** Brian Bauer, Raquel Norel, Alex Leow, Zad Abi Rached, Bo Wen, Guillermo Cecchi

**Affiliations:** 1 Department of Psychology, University of Georgia, Athens, GA, United States; 2 Digital Health, IBM Research, New York, NY, United States; 3 Department of Psychiatry, University of Illinois Chicago, Chicago, IL, United States; 4 Department of Biomedical Engineering and Computer Science, University of Illinois Chicago, Chicago, IL, United States; 5 College Louise Wegmann, Beirut, Lebanon

**Keywords:** natural language processing, explainable AI, suicide, mental health disorders, mental health disorder, mental health, social media, online discussions, online, large language model, LLM, downstream analyses, trauma, stress, depression, anxiety, AI, artificial intelligence, explainable artificial intelligence, web-based discussions

## Abstract

**Background:**

Rates of suicide have increased by over 35% since 1999. Despite concerted efforts, our ability to predict, explain, or treat suicide risk has not significantly improved over the past 50 years.

**Objective:**

The aim of this study was to use large language models to understand natural language use during public web-based discussions (on Reddit) around topics related to suicidality.

**Methods:**

We used large language model–based sentence embedding to extract the latent linguistic dimensions of user postings derived from several mental health–related subreddits, with a focus on suicidality. We then applied dimensionality reduction to these sentence embeddings, allowing them to be summarized and visualized in a lower-dimensional Euclidean space for further downstream analyses. We analyzed 2.9 million posts extracted from 30 subreddits, including r/SuicideWatch, between October 1 and December 31, 2022, and the same period in 2010.

**Results:**

Our results showed that, in line with existing theories of suicide, posters in the suicidality community (r/SuicideWatch) predominantly wrote about feelings of disconnection, burdensomeness, hopeless, desperation, resignation, and trauma. Further, we identified distinct latent linguistic dimensions (well-being, seeking support, and severity of distress) among all mental health subreddits, and many of the resulting subreddit clusters were in line with a statistically driven diagnostic classification system—namely, the Hierarchical Taxonomy of Psychopathology (HiTOP)—by mapping onto the proposed superspectra.

**Conclusions:**

Overall, our findings provide data-driven support for several language-based theories of suicide, as well as dimensional classification systems for mental health disorders. Ultimately, this novel combination of natural language processing techniques can assist researchers in gaining deeper insights about emotions and experiences shared on the web and may aid in the validation and refutation of different mental health theories.

## Introduction

Suicide rates have increased by 35% since 1999, and suicide remains a leading cause of death in the United States [1]. Despite concerted efforts, our ability to predict, explain, or treat suicide risk has not significantly improved over the past 50 years [2,3]. Thus, a top public health priority is understanding factors that contribute to suicide risk. Recent meta-analytic work, comprising suicide risk factor research over the past 50 years (using 365 studies), found that no single set of risk factors (eg, mood disorders or impulsivity) accurately predict future suicidal thoughts and behaviors [2]. Past risk factor studies have been limited by (1) potential sampling biases (eg, overrepresentation of clinical populations), (2) structured clinical interviews and surveys, and (3) laboratory-based (rather than naturalistic) settings. With the rapid increase in the use of web-based platforms, such as Reddit, people experiencing mental health symptoms have new outlets for sharing experiences, seeking support, and engaging in discussion regarding their mental health. Platforms such as Reddit provide unique opportunities for studying the experiences and perspectives of individuals at risk of suicide in the context of other mental pathologies and stressors [4-6]. To overcome previous limitations in suicide risk factor research, this study aims to analyze posts from a web-based community dedicated to providing support for individuals in crisis (ie, the r/SuicideWatch subreddit), to involve individuals who may not present for mental health studies or disclose their suicide risk and to obtain more nuanced insights into suicidality from the naturalistic and open-ended nature of anonymous web-based forums.

Understanding the factors contributing to suicidality is crucial for developing effective prevention strategies and interventions. Prominent theories of suicide—such as the Interpersonal Theory of Suicide (ITS) [7], Three-Step Theory (3ST) [8], and Integrated Motivational–Volitional (IMV) model [9]—are referred to as “ideation-to-action” frameworks. These theories attempt to explain how people develop suicidal ideation and transition to suicidal behaviors. Several common variables among ideation-to-action frameworks include feeling disconnected and burdensome to others, feelings of entrapment and hopelessness, and factors that may increase peoples’ capability to die by suicide (eg, traumatic experiences). Similar to many other psychological theories, they have been developed through researchers observing data patterns and testing their hypotheses (mainly) through self-reported survey data. Analyzing linguistic patterns in Reddit posts may provide an avenue for suicide theory exploration, confirmation, and refutation for these ideation-to-action frameworks, which could significantly impact future and existing intervention targets and assessment practices.

The use of natural language processing (NLP) with machine learning (ML) to gain new insights into mental health topics has increased dramatically over the last decade [10]. In mental health, this approach has mainly been used to confirm existing hypotheses through extracting meaning from texts (NLP) and then classifying these extractions (ML); however, this combination approach can be equally useful for exploration and discovery [10]. Specifically in suicide research, NLP and ML have primarily been used to help improve the accuracy of suicide risk identification [11]. However, NLP combined with ML is less frequently used in both mental health and suicide research to derive theoretical perspectives. Newer large language models (LLMs) that use Bidirectional Encoder Representations from Transformers (BERT) allow researchers to capture more complexities in human language than previous approaches, which are ideal for discovery as well as for testing directional hypotheses. Furthermore, the recent advances in explainable artificial intelligence (XAI) can be applied to NLP to help improve transparency, trustworthiness, and understanding of results in the context of mental health [12]. Using these modern techniques in tandem may help provide critical insights into suicide risk. The primary aim of this study is to analyze the content of posts from the r/SuicideWatch subreddit as well as mental health–related and non–mental health–related subreddits, with the goal of contributing to our understanding of suicide risk to ultimately improve prevention strategies and interventions. Although there are several other anonymous platforms available for individuals to discuss suicide, we chose Reddit due to the size of its userbase (over 400,000 members), which may aid the generalizability of our findings, and for practical reasons—namely, data availability. For this, we used LLMs to produce numerical representations of these posts—called *embeddings* [13]—which may reveal *unique* suicidality linguistic patterns; we also used recent developments in generative LLMs [14] and XAI [15], which turn abstract embeddings into natural language text, to identify connections with theories of suicidal behavior.

Our study was primarily data driven, as we used *generic* LLM embeddings of the posts and only applied theoretical constructs for post hoc interpretation. This means that the numerical representation of the posts was based on how the sentences in them are related to sentences in very large text corpora used for training the LLM that cover vast swaths of topics.

## Methods

### Ethical Considerations

Reddit users are made aware that their posts are publicly accessible through Reddit’s Terms and Conditions. No personal identifying information (eg, names, locations, or IP addresses) were collected. Further, none of the authors participated in any discussions; thus, it was not necessary to inform users that their posts may be used for research. Because the collected data set is publicly available and already deidentified, the University of Georgia Human Subjects Office reviewed the submission and assigned a determination of “Not Human Research.”

### Data Procurement, Selection, and Preprocessing

We downloaded posts from a list of subreddits from October 1 to December 31, 2022, and from October 1 to December 31, 2010, using The-Eye.eu [16], which contains an archive of Reddit’s full submission until December 2022. We used Python (Python Software Foundation) to process the data. Posts that were removed or deleted were not used. Empty entries or entries containing just a “?” were not considered. We analyzed 16 subreddits related to mental health and 14 subreddits not related to mental health to serve as controls. In Table 1, we compared the number of posts and words per post by 2 subreddit groups (mental health and controls). We saw that the number of posts was similar in both groups, but the range and variability (ie, SDs for the number of words and words per post) were much higher in the mental health subreddits. We noted that for the 2010 data, in general, the posts were much longer than those from 2022 data; in the 2022 data, some of the posts were just a single word (eg, “Pls,” “Yuh,” and “Help”), whereas for older data (2010), the posts were much longer.

**Table 1.** Statistical descriptors of the 16 subreddits related to mental health and the 14 subreddits used as controls.

Subreddits and statistics		2010 data		2022 data	
		Mean (SD)	Range	Mean (SD)	Range
**Mental health**					
	Posts per subreddit	246 (225)	14-785	182,000 (240,000)	15,900-928,000
	Words per subreddit	409,000 (415,000)	42,000-1,600,000	34,700,000 (48,800,000)	2,700,000-189,000,000
	Words per post	1700 (446)	1000-3000	183 (31)	112-225
**Controls**					
	Posts per subreddit	—^a^	—	195,000 (243,000)	9700-831,000
	Words per subreddit	—	—	21,900,000 (28,300,000)	1,500,000-95,000,000
	Words per post	—	—	114 (27)	72-163

a Not applicable.

### Linguistic Analysis and Interpretation

#### Overall Approach

After obtaining the posts, we followed these steps: (1) represented the posts in the latent space of LLM embeddings; (2) computed a representation for each subreddit, averaging all its posts; (3) computed different metrics of the structure of the subreddits in the embedding space; and (4) applied interpretation techniques to these structural metrics to obtain insights about the relationship between suicidality and other self-identified groups.

#### LLM Embeddings

Semantic text embedding is an NLP technique used to represent the meaning of text in numerical form. It accounts for the context of words or phrases rather than just their individual representations. By using advancements in text embedding, we used a more precise method in NLP using BERT [17]. Text embedding assigns a numerical vector to each text, enabling texts with similar contexts to be closer in the vector space. This allows us, using mathematical tools, to better understand and analyze the semantic similarities and differences between different texts. To represent a subreddit, we compute the centroid (average) of the embeddings of all the posts assigned to it. As mentioned above, these embeddings are *unbiased*: we did not use the metadata related to the subreddit provenance nor applied any theory-driven construct [18].

#### Subreddits Structure

To obtain a measure of similarity between subreddits, for each post, we computed a “linguistic label”—the label of the nearest centroid in the embedding space. Then, for each subreddit, we computed the percentage of posts that were assigned to any “linguistic label,” including the original label; the proportion of posts “linguistically assigned” to a subreddit other than the original one is a measure of the similarity between them.

#### Hierarchical Clustering

Hierarchical clustering operates on the principle of iteratively merging the closest pair of clusters, where the definition of “closeness” varies according to different linkage criteria. We used the Ward linkage method, where the distance between 2 clusters is the increase in the summed square distance from each point to the centroid of its cluster after merging the clusters. A dendrogram (a tree-like diagram) visually represents the process and results of hierarchical clustering. Each leaf corresponds to a data point, and branches represent the successive merging of clusters, with the height of each merge proportional to the distance between the combined clusters. By examining a dendrogram, one can intuitively grasp the data’s structure and decide on an appropriate number of clusters by cutting the tree at a specific height. This visual tool aids in interpreting the complex relationships and nested structures within the data, offering insights into the underlying patterns and groupings. We performed hierarchical clustering on the embedding representative of the subreddit, that is, the average of all the embedding vectors of the subreddit.

#### Dimensionality Reduction

We factorized the subreddits centroids in the embedding space using singular value decomposition (SVD). We analyzed the relative location of the subreddits in the reduced representation of the first 3 SVD components, understood as latent semantic dimensions.

#### Generative LLM

We used a generative LLM to obtain insights into the latent patterns of the SVD components. In a Jupyter Notebook environment, we configured the *Langchain* library to use the GPT-4-0613 model (OpenAI) [14] with a zero-temperature setting, ensuring deterministic outputs for consistency in interpretation. We used the *ConversationChain* module coupled with *ConversationBufferMemory* to facilitate an interactive and memory-aware dialogue with GPT-4. This setup enabled us to iteratively query the model with our prompts and data, ensuring a contextually rich and coherent analysis of the subreddit postings. The use of a verbose mode in the conversation setup provided detailed logging of the model’s responses, further aiding in the transparency and traceability of our analytical process. We identified posts with the top and bottom 5 embedding positions in the first 3 dimensions of SVD projections, totaling 30 extreme postings.

#### XAI Techniques

**Figure 1.**
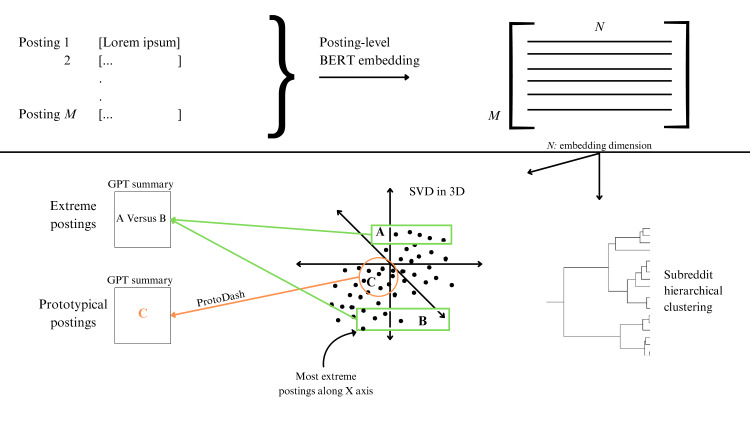
Schematic description of the processing workflow in this paper. All Reddit postings were first fed into BERT to yield posting-level embeddings in a high-dimensional space, followed by dimensionality reduction (into 3D) using SVD. This procedure allows us to extract prototypical postings (using ProtoDash) as well as extreme postings along each of the 3 SVD axes, which were then fed into ChatGPT for semantic interpretations. Last, we also performed hierarchical clustering to recover the relational structure between different subreddits. BERT: Bidirectional Encoder Representations from Transformers; GPT: Generative Pre-trained Transformer; SVD: singular value decomposition.

XAI techniques are designed to provide insights into the factors or features that contribute to an artificial intelligence system’s outputs, allowing users to understand and validate the reasoning behind those decisions. With this in mind, we use ProtoDash [15], a technique used to choose representative examples that effectively represent the overall distribution of a data set, to help with the interpretation of the model. We fed the LLM embeddings and the associated posts from the r/SuicideWatch subreddit to ProtoDash and asked for the 5 most representative posts in the data set. Those selected prototypes of the data set were then fed to ChatGPT, to help obtain insight into the data. Figure 1 shows a visual overview of the study methodology.

## Results

Unless explicitly indicated, we presented results on the 2022 data; the 2010 data were used to ascertain the stability of the structural features we determined with the most recent and larger data set.

### Verbosity in Mental Health Subreddits

**Figure 2.**
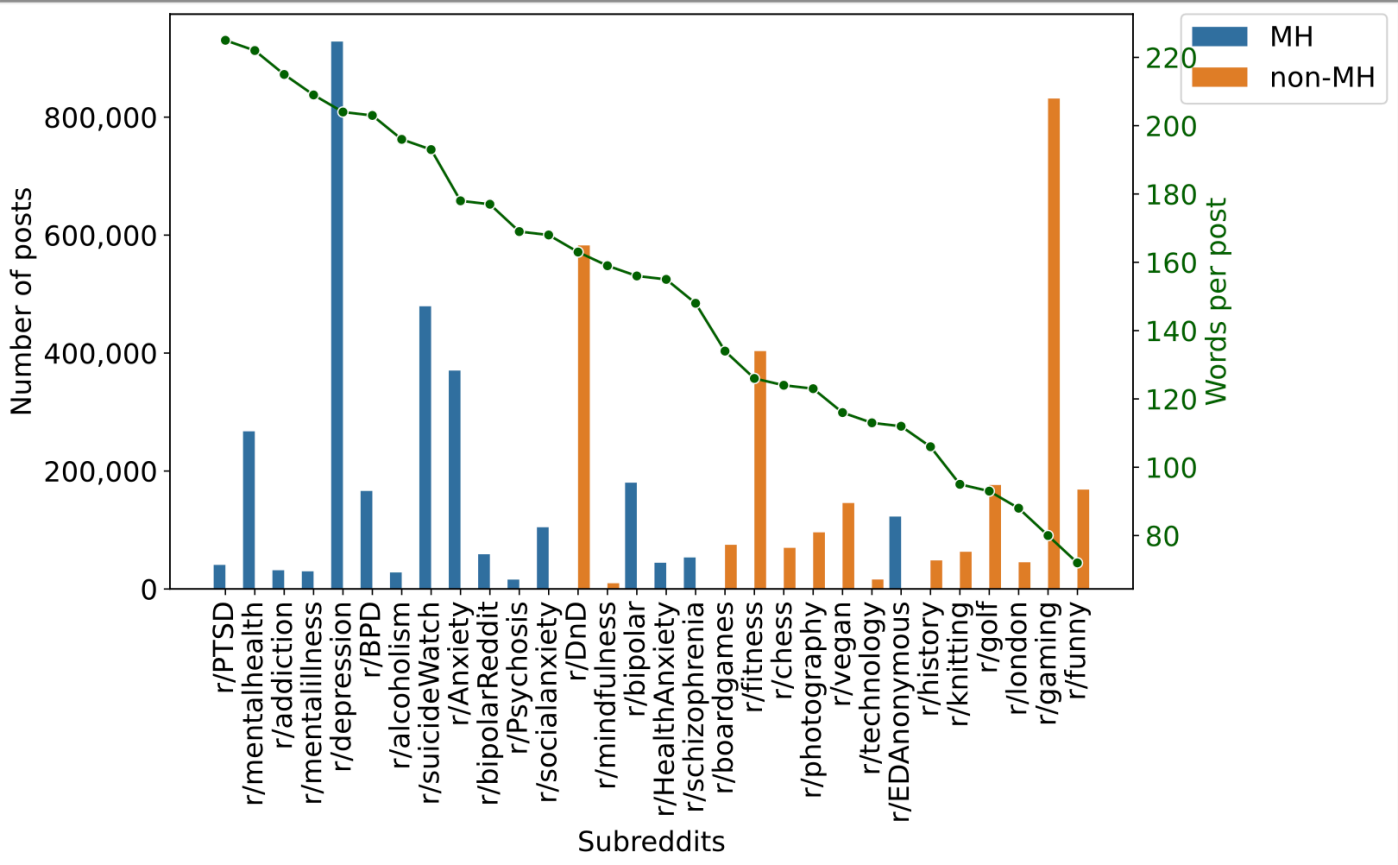
The total number of posts (bar heights) and verbosity (number of words per post; green line) of subreddits. The verbosity of MH subreddits (blue) is significantly higher than non-MH ones (orange). BPD: borderline personality disorder; DnD: Dungeons and Dragons; EDAnonymous: eating disorders anonymous; MH: mental health; PTSD: posttraumatic stress disorder.

The total number of posts and the verbosity (the average number of words per post in a subreddit) are shown in Figure 2. R/Depression is the most active of the mental health subreddits, and only behind the r/DnD (referring to Dungeons and Dragons) and r/gaming subreddits overall. The subreddits were sorted by verbosity (green line), which shows higher values for mental health posts as opposed to non–mental health posts. Moreover, within mental health subreddits, there are differences of more than 30% between the least verbose subreddits (r/schizophrenia and r/EDAnonymous [eating disorders anonymous]) and the high-verbosity subreddits (r/PTSD [posttraumatic stress disorder] and r/Depression).

### Structure of the Linguistic Embedding Space

**Figure 3.**
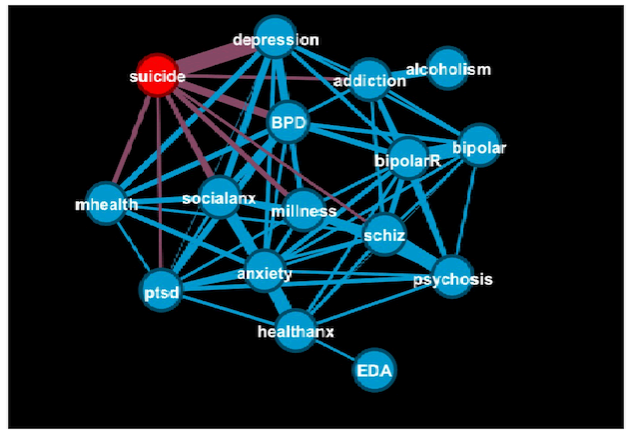
Proportion of posts from a given subreddit that are the “closest” to a different subreddit centroid. The r/SuicideWatch centroid is colored red, nodes connected to it are colored purple, and the rest of nodes are colored blue. The width of the edge is proportional to the number of posts that are the closest to each centroid. addition: r/addiction; alcoholism: r/alcoholism; anxiety: r/SocialAnxiety; bipolar: r/Bipolar; bipolarR: r/BipolarReddit; BPD: r/BPD (borderline personality disorder); depression: r/Depression; EDA: r/EDAnonymous (eating disorders anonymous); healthanx: r/HealthAnxiety; mhealth: r/MentalHealth; millness: r/mentalillness; psychosis: r/Psychosis; ptsd: r/PTSD (posttraumatic stress disorder); schiz: r/schizophrenia; socialanx: r/SocialAnxiety; suicide: r/SuicideWatch.

The plot in Multimedia Appendix 1 shows the measure of similarity to r/SuicideWatch for all subreddits. Almost half of the posts (234,406/479,321; 48.9%) from r/SuicideWatch were closer to their centroid than to any other centroid, with an additional 12.7% (60,854/479,321) being the closest to r/Depression, followed by r/BPD (borderline personality disorder) and r/SocialAnxiety. The extension of this approach to all subreddits is shown in Figure 3, which represents inter-subreddit similarities in the width of the links, thresholded at 7%. Besides the strong associations already present in Figure 2 (of r/SuicideWatch with r/Depression and r/BPD), there are associations of r/BPD with r/Depression; r/SocialAnxiety and r/PTSD; and several associations between the different anxiety-related subreddits, r/Psychosis and r/schizophrenia, the 2 bipolar subreddits, and r/addiction with r/alcoholism.

**Figure 4.**
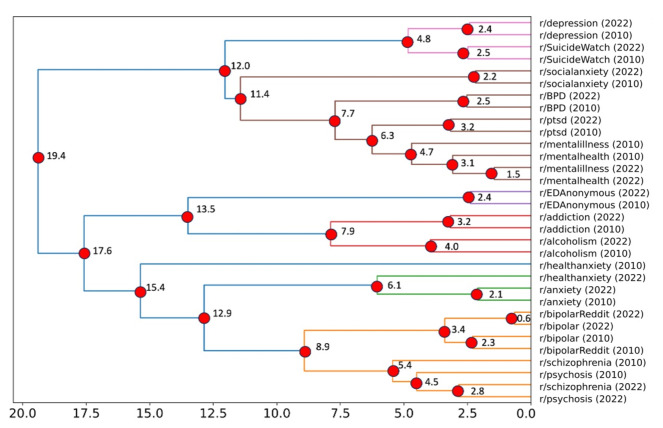
Dendrogram of mental health vectors, using Ward linkage of subreddit’s embeddings. The horizontal axis represents the linkage distance; clusters that combine at shorter distances are more similar to each other; as the distance grows, these clusters join together to form larger, more general categories. BPD: borderline personality disorder; EDAnonymous: eating disorders anonymous; PTSD: posttraumatic stress disorder.

The dendrogram in Figure 4 represents the result of the hierarchical clustering of the centroid coordinates for all the mental health subreddits in 2022 and 2010. The horizontal axis representing the linkage distance illustrates and supports the sequential merging of clusters. Clusters that merge at lower distances are more akin; as the distance increases, the clusters amalgamate into broader categories. The cluster with the shortest distance is grouping the subreddits r/BipolarReddit and r/Bipolar. The following cluster consists of the subreddits that discuss mental illness (r/mentalillness) and mental health (r/MentalHealth). The third most similar cluster is formed by r/Psychosis and r/schizophrenia. R/Anxiety and r/HealthAnxiety cluster together (green), as do the subreddits regarding addiction (r/alcoholism and r/addiction; red). Importantly, the clustering of the 2022 and 2010 subreddits were highly consistent, supporting the notion that the posts by the different subreddits revolve around the same topics related to the mental health condition they identify with.

### Interpretation of the Linguistic Embedding Space

**Figure 5.**
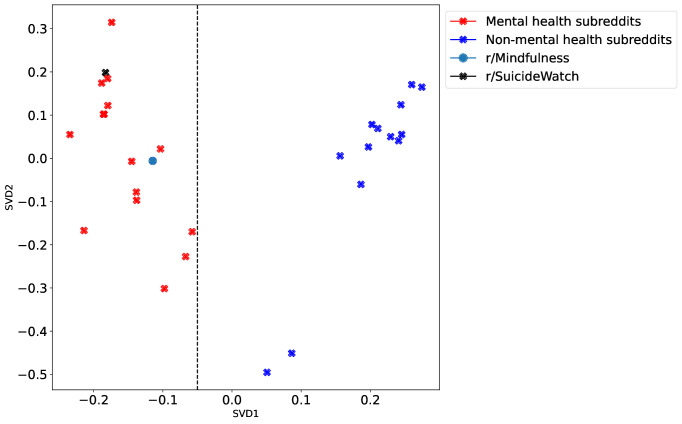
SVD factorization of the embedding space. With the exception of r/Mindfulness, all non-mental health subreddits have positive value on the first SVD component, whereas all mental health subreddits have negative value on the first SVD component. SVD: singular value decomposition.

To obtain insights into the meaning of these topics, we performed a SVD factorization of the embedding space and used interpretation techniques on the resulting factors. Figure 5 shows the relative location of all the subreddits in the space determined by the first 2 SVD components. With the exception of r/Mindfulness, which is close to the mental health subreddits, there is a clear separation of classes along the SVD1 dimension. We also observed that r/SuicideWatch was ranked the second highest in SVD2, suggesting that this dimension may contain patterns relevant to suicidality.

**Figure 6.**
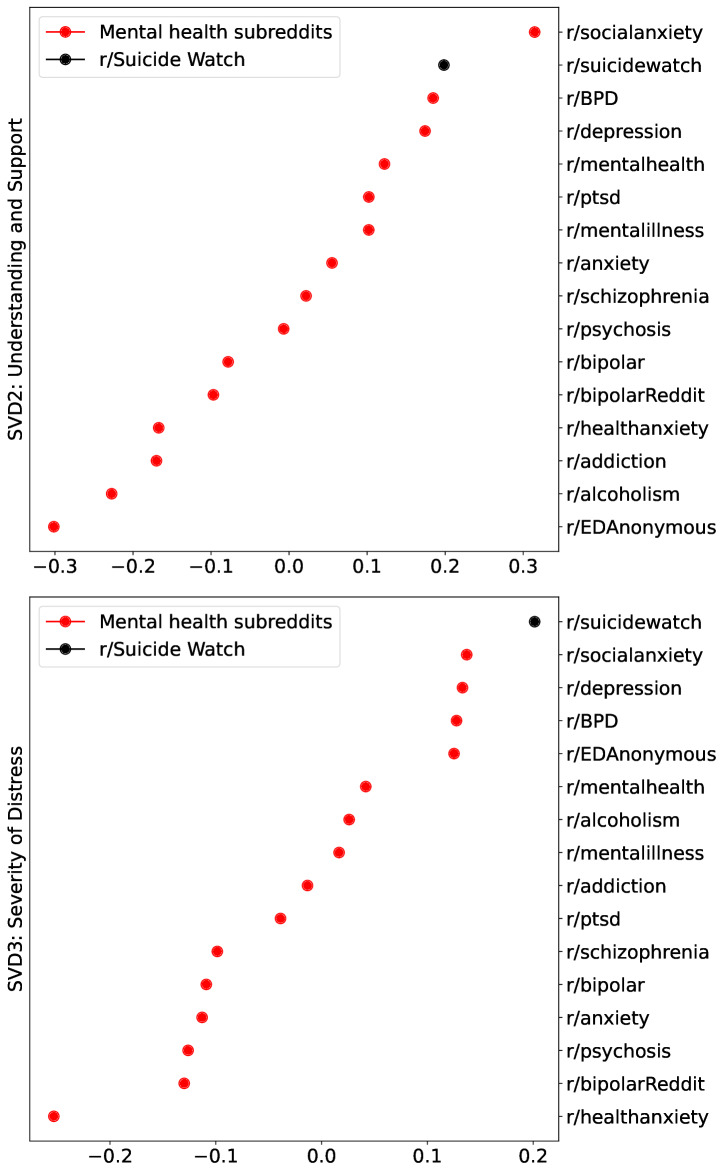
SVD factorization of the embedding space for mental health subreddits. Centroids (representing each subreddit) are sorted from lowest to highest value on the second SVD factor in the top panel and on the third SVD factor in the bottom panel. On both panels, that is, for the second and third SVD components, the 4 extreme values correspond to r/SuicideWatch, r/SocialAnxiety, r/Depression, and r/BPD. BPD: borderline personality disorder; EDAnonymous: eating disorders anonymous; PTSD: posttraumatic stress disorder; SVD: singular value decomposition.

The result of this procedure is presented in Table 2, which can be summarized by the following labels and directionalities: SVD1, *Emotional Well-Being: Despair to Resilience*; SVD2, *Seeking Understanding and Support: Closing In to Reaching Out*; and SVD3, *Severity of Distress: Low to High*. Using these axis interpretations, we mapped the results from the second and third SVD projection only for the mental health subreddits, for better visualization and interpretation; Figure 6 shows only mental health cases, which fall within the left portion of Figure 5. A prominent feature of the 2 plots is that r/SuicideWatch mapped onto the high end of the *Understanding and Support (Reaching Out)* dimension, as well as on the high end of the *Severity of Distress (High Distress)* dimension in a completely data-driven way.

- Axis label prompt: “Identify the name of the axis spanned by these two groups of extreme postings given that if one extreme is hot, the other extreme is cold, then the axis is temperature.”
- High values prompt: “What are the similarities among the top (highest values on the axis) five postings.”
- Low values prompt: “What are the similarities among the bottom (lowest values on the axis) five postings.”

**Table 2.** Interpretation based on GPT-4 for prompts for the main 3 singular value decomposition (SVD) axes. Text from GPT-4 has been edited for grammar and clarity.

Values	Interpretation and axis label		
	SVD1, Emotional Well-Being: Despair to Resilience	SVD2, Seeking Understanding and Support: Closing In to Reaching Out	SVD3, Severity of Distress: Low to High
High	Struggle and resilience Experiencing significant anxiety, depression, and life changes Learning to trust themselves and their abilities to handle their situations Actively seeking help and trying to find ways to manage their mental health (eg, therapy, self-care, and relaxation techniques) Making progress toward self improvement and happiness	Seeking guidance or advice Desire for information, advice, or validation Reaching out for insights, recommendations, or shared experiences This emotion is intertwined with feelings of uncertainty, curiosity, and a desire for understanding or improvement	More intense and distressing content Severe mental health struggles, including suicidal thoughts and feelings of extreme despair Darker tone and more desperate
Low	Narrative of struggle and despair Feelings of sadness and emptiness Express a sense of hopelessness about their situation Previously sought help (eg, therapy and medication) but feel that these methods have been ineffective	Feelings of internal conflict, frustration, and being overwhelmed by personal challenges Deep sense of pain stemming from mental health struggles, physical health issues, or personal insecurities There’s a recurring theme of individuals grappling with their emotions and seeking understanding, validation, or support	Moderate distress and struggles with mental health Discusses personal experiences with mental health struggles, seeking help, and dealing with anxiety and social situations

To complement our previous analyses that relied on extreme postings, here, we instead leveraged prototypical or representative postings within the r/SuicideWatch subreddit. To this end, we used ProtoDash to select the top 3 most representative postings, which were then fed into GPT-4 with the prompt of “Are these postings in line with current theories about suicide and suicidal ideation?” The following summarizes GPT-4’s response:

The first post describes feelings of hopelessness, despair, and anxiety, with a clear intent to commit suicide. The second post, despite stating a lack of suicidal intent, expresses chronic emotional distress, a lack of enjoyment in life, and recurring thoughts of wanting to die, which may actually be signs of suicidal ideation. The third represents a state of severe emotional distress, feelings of hopelessness, despair, loneliness, and a sense of being misunderstood and neglected; it also mentions previous suicide attempts, a plan for a final attempt, and mentions hearing voices, which could suggest a psychotic disorder.

These posts are consistent with current theories of suicidality. The ITS, for example, posits that individuals are more likely to die by suicide when they have both the desire to die, often stemming from feelings of burden and social isolation, and the capability to do so, often developed through previous exposure to painful or fear-inducing experiences.

## Discussion

### Principal Findings

We combined analytic and interpretability techniques to study linguistic contents in large numbers of postings derived from the r/SuicideWatch subreddit in relation to other mental health subreddits, as well as select non–mental health subreddits. This allows us to better understand, both qualitatively and quantitatively, how suicidal ideation linguistically presents in popular social media sites (Reddit). Our results offer new insights into the emotional and thematic content shared by individuals at risk of suicide on Reddit.

First, by applying GPT-4 to postings spanning the top 3 axes in our dimensionally reduced embeddings across all subreddit postings, we were able to determine the linguistic meanings of these axes, which describe generally what people tend to discuss on anonymous web-based mental health forums, including suicide-related discourse. These axes were (1) resilience versus despair, (2) validation versus advice, and (3) high versus low distress. R/SuicideWatch was characterized by narratives of struggles and despair (eg, hopelessness and previous treatment being ineffective) and showed the highest values of nearly all mental health subreddits for seeking advice (more so than validation) and high levels of distress. R/SuicideWatch posts also had the highest values for seeking guidance and advice—just above r/BPD and r/Depression and just below r/SocialAnxiety. These specific communities and those with associated disorders or phenomena may seek advice or guidance (ie, practical solutions) more than others because of their chronicity, recurrence, and historical difficulty to treat. For example, spanning across 50 years of intervention research for suicidal thoughts and behaviors, recent meta-analytic evidence shows that overall intervention effects are small regardless of the intervention or suicide-related outcome studied [3]. Further, although effective treatments are available, when left untreated, social anxiety and BPD are often chronic conditions [19,20], and depressive disorders are highly recurrent [21]. Individuals on Reddit may therefore be searching for additional solutions, potentially because past treatment engagement was not adequately effective in reducing their symptoms. Last, regarding distress, our study is in line with previous studies examining suicide-related social media posts that characterizes these postings as indicating high levels of distress [22].

More generally, these 3 axes exemplify broad common themes during clinical appointments, with people sharing messages of *hope and resilience* or *despair and hopelessness* (referred to as “Emotional Well-Being” by GPT-4). Similarly, individuals on mental health subreddits mention a desire for *validation or support* and *problem-solving or solutions*, both of which are core to several effective psychosocial treatments. Last, subjective distress represents a core component of what helps define a mental health disorder [23] and varies widely from disorder to disorder as well as individually, as indicated by the values along this axis. Together, these axes may indicate that a therapy-like process naturally occurs in Reddit communities, where posters provide messages of despair or resilience with different degrees of distress and are searching for validation and solutions to their experiences.

Using ProtoDash in conjunction with GPT-4, we extracted and summarized common thematic and emotional contents from r/SuicideWatch postings. Results support the central variables within contemporary theories of suicide for explaining why the desire for suicide develops. Here, the 3 most prototypical posters predominantly wrote about feelings of disconnection, burden, hopelessness, desperation, resignation, and trauma. The ITS, 3ST, and IMV theories of suicide all state that disconnection (eg, thwarted belongingness), perceived burdensomeness, and feeling that their issues are intractable (eg, entrapment, hopelessness, and resignation) are necessary elements for developing a desire to die by suicide. Further, desperation—having a deep sense of despair, feeling overwhelmed, and lacking the ability to improve current conditions—is consistent with psychache [24] and the recently proposed diagnostic criteria for acute suicide conditions (eg, suicide crisis syndrome [25]), each of which cite despair as a core criterion for the development of suicidal ideation. Last, experiencing trauma (broadly defined) was frequently discussed in postings. Experiencing traumatic events is not posited as a necessary and sufficient condition for the development of suicidal ideation, but it has been put forth as a contributor for why people become capable of dying by suicide [7-9]. However, previous meta-analytic work has correlated traumatic experiences such as abuse with future suicidal ideation [2], and if the term is taken colloquially (ie, negative life stressors), it may contribute to theoretical constructs such as defeat or humiliation [9,25].

Next, we identified linguistically defined natural groupings among mental health subreddits. Our results seemed to suggest three different clusters: (1) r/SuicideWatch, r/Depression, r/MentalHealth, r/BPD, and r/Social Anxiety; (2) r/Psychosis, r/schizophrenia, and r/Bipolar; and (3) r/alcoholism, r/EDAnonymous, and r/addiction (see Figures4  5). However, the 3 clusters mentioned above appear to (largely) support the superspectra put forth by the Hierarchical Taxonomy of Psychopathology (HiTOP)—a recent statistically driven diagnostic classification system for mental disorders [26]—compared to traditional clusters found in the *Diagnostic Statistical Manual of Mental Disorders, Fifth Edition* [23]. The three clusters mentioned above generally correspond to the following HiTOP spectra: (1) Internalizing Disorders; (2) Psychotic Disorders; and (3) Disinhibited Externalization Disorders. However, other mental health subreddits such as r/HealthAnxiety, r/Anxiety, r/mentalillness, and r/PTSD did not map as neatly onto any 1 dimension. In contrast with HiTOP, r/BPD was more aligned with internalizing disorders than externalizing disorders in this study. Similarly, while eating disorders (r/EDAnonymous) and substance or alcohol use disorders (r/addiction) are classified under different HiTOP spectra (Internalizing Disorders and Disinhibited Externalizing Disorders, respectively), their strong phenotypic associations and comorbidities are well documented in the literature (see a recent study examining their shared genetic risks [27]). Taken as a whole, our results also support a more nuanced and dimensional view of HiTOP spectra (eg, internalizing vs externalizing disorders), where posters in r/BPD are more often discussing unsatisfying relationships, feelings of emptiness, desires for self-harm, anger, and other cognitive criteria rather than solely discussing externalizing behaviors (eg, physical fights and risky behaviors) that are central to the BPD HiTOP spectra (ie, externalizing disorders).

As indicated by the hierarchical structure of the dendrogram in Figure 4, the linguistic features in r/SuicideWatch have substantial overlap with postings in r/Depression, r/BPD, and r/SocialAnxiety. This is likely due to suicidal ideation being a symptom of depression and suicidal (and parasuicidal) behaviors, which are common features of BPD [23]. Regarding the (somewhat unexpected) linguistic similarities between postings from r/SuicideWatch and r/SocialAnxiety, we posit that they are likely driven by mentioning social and interpersonal issues (eg, loneliness), which are common among both. These similarity findings may help serve as additional validity for our results and may have implications for recently proposed diagnostic criteria for suicide-related thoughts and behaviors, such as acute suicide affective disorder [28] and suicide crisis syndrome [25], that highlight abrupt or accentuated feelings of social disconnection or social withdrawal as an indicator of suicide crisis risk.

From Figure 6, we note in the top panel that r/BPD had the third highest value on the axis for “Understanding and Support,” with r/SuicideWatch being the second highest. In the bottom panel, r/BPD has the fourth highest value on the axis for “Severity of Distress,” next to r/Depression, whereas r/SuicideWatch had the highest value. Individuals with BPD are at a notably higher risk of suicide, with commonalities between the 2 including impulsivity, intense emotional dysregulation, and chronic feelings of emptiness. These individuals often struggle with unstable interpersonal relationships and heightening feelings of loneliness and rejection, which can trigger suicidal thoughts and behaviors. Additionally, a significant proportion of those with BPD have a history of trauma and may have co-occurring mental health disorders, such as depression or anxiety, further exacerbating the risk. The prevalence of self-harm behaviors in individuals with BPD, although not always indicative of suicidal intent, is also a critical risk factor [29-32].

Last, the representative posts selected by ProtoDash and interpreted by ChatGPT showed that r/SuicideWatch discussions emphasized feelings of empathy, support, understanding, and gratitude. In addition, ChatGPT found that typical replies offered messages of hope that things will improve and encouraged seeking help, traveling to beautiful places or to find solace, and connecting with nature to find “self love.” Many of these actions (eg, empathy, support, understanding, and encouraging help-seeking behavior) are what professional organizations (eg, National Suicide Prevention Lifeline and American Foundation for Suicide Prevention) advocate friends, family, and communities provide to individuals in crisis. Although some of the proposed suggestions do not have an evidence base for being effective in reducing suicidal desire (eg, traveling to beautiful places), survivors of suicide or individuals with lived experience (eg, other members of r/SuicideWatch) may provide additional perspectives that can be helpful when used alongside evidence-based therapies and interventions [33]. Overall, these findings indicate that some web-based communities, such as r/SuicideWatch, could be a source of support for many individuals experiencing suicidal thoughts and can act in accordance with the suggestions put forth by several professional suicide prevention organizations.

### Limitations and Future Directions

We note a few limitations of our study. First, the data were limited to a 3-month period, which may not be sufficient to fully capture the range of experiences and emotions expressed in the r/SuicideWatch subreddit. Additionally, instead of more broadly looking into other social media platforms, the study focused solely on Reddit, and thus, the findings may not generalize to other web-based platforms. Future research could expand the time frame of data collection; explore other web-based platforms; and integrate additional data sources, such as user comments, to provide a more comprehensive understanding of web-based expressions of suicide risk. Last, the results from this study could not be validated against external criteria such as established measures of suicide risk or clinician judgment, potentially limiting the credibility of our findings. Future studies could incorporate multiple perspectives to help understand the accuracy and reliability of the extracted thematic interpretations.

### Conclusion

In conclusion, we used a novel combination of NLP techniques to detect and interpret linguistic patterns of mental health subreddits to better understand how suicidal ideation presents in web-based communities. LLM embeddings allowed for a nuanced analysis of subreddit content that revealed unique patterns and shared themes that are specific to suicide-related content. Further, dimensional reduction revealed latent dimensions of mental health discussions and helped identify relationships between various subreddits. Last, we used generative LLM for XAI to gain deeper insights into the emotions and experiences of individuals posting about suicidal thoughts. Our results supported contemporary theories of suicide. Our study highlights the potential use of web-based linguistic patterns as valuable data sources to better understand mental health disorders and suicidality.

## Supplementary material

10.2196/57234 Multimedia Appendix 1 Proportion of posts from a given subreddit “closest” to the r/SuicideWatch centroid.

## Abbreviations

3ST: Three-Step Theory
BERT: Bidirectional Encoder Representations from Transformers
BPD: borderline personality disorder
EDAnonymous: eating disorders anonymous
HiTOP: Hierarchical Taxonomy of Psychopathology
IMV: Integrated Motivational–Volitional
ITS: Interpersonal Theory of Suicide
LLM: large language model
ML: machine learning
NLP: natural language processing
PTSD: posttraumatic stress disorder
SVD: singular value decomposition
XAI: explainable artificial intelligence
