# Supplementary figures and images for: Using Large Language Models to Understand Suicidality in a Social Media–Based Taxonomy of Mental Health Disorders: Linguistic Analysis of Reddit Posts
Source: JMIR Ment Health. 2024 May 16;11:e57234. doi: 10.2196/57234 (PMC11112053; doi:10.2196/57234)

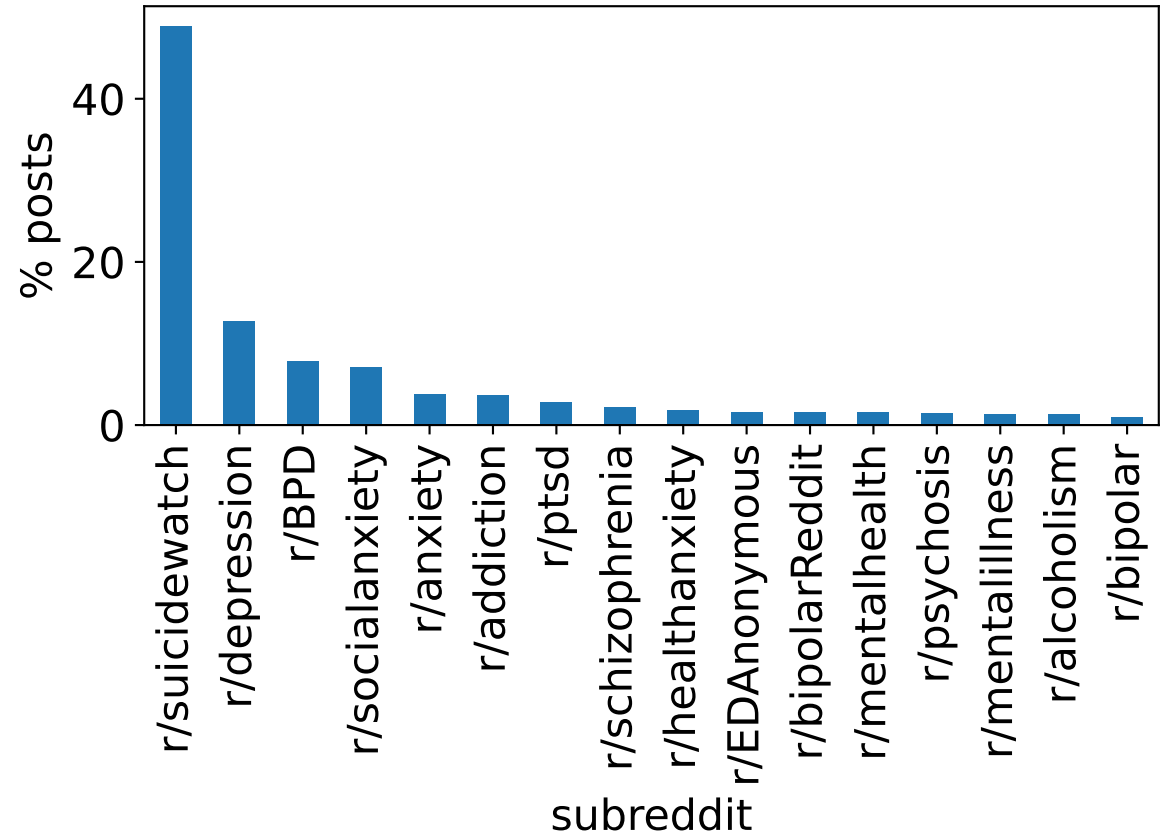

Supplement: Multimedia Appendix 1 [file mental-v11-e57234-s001.pdf]
